# The impact of the sports industry on the development of the health industry: an exploration of mediating and threshold effects

**DOI:** 10.3389/fpubh.2025.1691241

**Published:** 2025-11-18

**Authors:** Shutong Zhao, Haiyan Huang

**Affiliations:** 1 School of Economics and Management, Shanghai University of Sport, Shanghai, China; 2 College of Physical Education, West Anhui University, Lu'an, China; 3 School of Management, Beijing Sport University, Beijing, China

**Keywords:** health industry, sports industry, fixed effect, mediating effect, threshold effect, influence mechanism

## Abstract

**Introduction:**

The sports and health industries play a crucial role in fulfilling individuals' aspirations for an enhanced quality of life and in supporting the development of a Healthy China.

**Methods:**

To explore the impact mechanism of the sports industry (SID) on the development of the health industry (HID), this study employed a rigorous empirical framework utilizing panel data from 30 provinces in China over the period 2014–2023. Using fixed-effects and mediation-effect models, this study examined the influence mechanism and identified the key pathways through which the sports industry impacts the development of the health industry.

**Results:**

The results demonstrate that the sports industry positively influences the development of the health industry. This conclusion remains robust across a series of robustness tests. The heterogeneous analysis reveals regional variation in the impact of the sports industry on the health industry development. Among the regions, the sports industry has the strongest promotional effect on health industry's development in the western regions, followed by the eastern and central regions. Conversely, the impact in the northeastern regions is relatively negligible. Mediation effect analysis reveals that the sports industry can effectively stimulate the development of the health industry by significantly increasing R&D intensity. Furthermore, testing the threshold effect indicates that the impact of the sports industry on the health industry exhibits a double threshold and non-linear diminishing marginal effects.

**Discussion:**

This study contributes to a comprehensive understanding of the sports industry's impact on the health industry. Based on these findings, we proposed a series of targeted recommendations.

## 1 Introduction

Health is a fundamental and vital human need. The Coronavirus disease 2019 (COVID-19) pandemic, in particular, highlighted that everyone is vulnerable to diverse and uncertain health risks (1). The demand for good health has led individuals to be more willing to purchase health services and health-promoting products. As a result, “investing in health” has become a growing consumer trend. The Healthy China 2030 Planning Outline underscores the need to actively advance the amalgamation of health with sports-related activities, including fitness and recreational pursuits, while cultivating the growth of sports rehabilitation and the health culture sector. In 2016, the State Council Office issued “Guiding Opinions on Accelerating the Development of Fitness and Leisure Industries,” highlighting the need to integrate fitness and leisure with sectors such as culture, senior care, education, health, agriculture, forestry, and transportation. In the context of economic restructuring and the Healthy China initiative, the sports industry (SID) has received considerable attention as a vital catalyst and growth driver for advancing the health industry (HID) (2).

The state's vigorous promotion of the sports industry can rectify the shortcomings in China's existing medical and health system and meet the diverse health needs of its population (3). As the differentiation and personalization of health consumption demands among Chinese residents escalate (4), the sporting goods industry must offer health products for disease prevention and chronic disease management to individuals across their entire life cycle (5). To shift health protection from reactive to proactive, certain residents require individualized physical activity in addition to medical interventions. This can change the passive acceptance of medical care into an active pursuit of health (6). The swift increase in demand for healthcare has also prompted the restructuring of numerous value chain components, allowing for the development of new health goods to satisfy consumer needs and the ongoing improvement of healthcare services (7). The convergence of sports and health is set to materialize (8), presenting significant market potential and a substantial pool of untapped customers, indicating robust growth momentum. Guided by national policies, the convergence of these two industries has gathered market capital, resulting in diverse new business models within the “sports + health” sector (9), including sports health management services (encompassing fitness and leisure services, sports health consultation and management, sports health tourism, and sports healthcare for the older adults, among others). The development of the sports industry enables the integration of diverse resources to collectively promote the health of the entire population (10). Addressing varying health requirements simultaneously fosters new growth opportunities, offering multifaceted support for superior economic development (11). Therefore, conducting a comprehensive investigation into the influence of the sports industry on the development of the health industry holds significant theoretical and practical importance.

To answer the aforementioned questions, this study examines the influence of the sports industry on the health industry, aiming to rigorously quantify its impact on health industry development and to analyze the underlying mechanisms. This study highlights the following significant contributions. First, we provide scientific data and empirical evidence about the promotion of the health industry by the sports industry. Second, we investigate the precise mechanisms by which the sports industry impacts the health industry, incorporating R&D intensity as a mediating variable, and delineating the transmission pathway “sports industry - R&D intensity - health industry” to elucidate the influence of the sports industry on the health industry. Third, by accounting for regional heterogeneity, this study examines the promotional influence of the sports industry on the health industry in a regional context. This is the first study that employs empirical data to explore the threshold effect between the sports and health industries.

## 2 Literature review

The health industry, which includes the healthcare, life sciences, public health, and wellness sectors, is entering a phase of rapid development (12). Scholars have undertaken theoretical research on the health industry, examining its attributes, scope definition, and scale measurements (13), as well as the roles of the government and market in its development (14). Among the literature on the relationship between the health industry and other industries, Hu et al. (15) found that a highly developed pharmaceutical manufacturing industry can improve synergistic collaboration with the medical service industry. However, optimizing the structure of the pharmaceutical manufacturing industry may impede effective cooperation between the two industries. Wang et al. (16) investigated the coordination and regional evolution patterns between health resource distribution and economic development, employing a fixed-effects model to identify the factors driving their interaction. Yang et al. (17) used the coupling coordination degree model and a panel Tobit model to examine the coupling coordination between healthcare service supply and high-quality economic development. Deng et al. (18) employed a coupling coordination degree model and kernel density estimation to assess the spatial agglomeration impacts and spatial convergence characteristics of healthcare service provision and regional economic coupling growth. Overall, the literature demonstrates a rapidly expanding health industry, clarifies its conceptual boundaries and governance roles, and evidences inter-industry coordination dynamics, especially between pharmaceutical manufacturing and medical services, alongside marked regional heterogeneity and spatial agglomeration in the coupling of healthcare resources and economic development.

Since the 1990s, scholars have increasingly focused on the interplay between the sports industry and other industries (19), participating in vigorous discourse concerning the linkages among sports, tourism, the ecological environment, and the digital economy. Cheng et al. (20) employed the entropy approach, coupling coordination degree, kernel density estimation, and gray relational analysis to assess the coupling and coordination between the sports and tourism industries, along with their driving variables. The findings demonstrated a high degree of coupling between the sports industries and tourism industries during their development. Wang et al. (21) utilized a coupling coordination degree model and exploratory spatial data analysis to investigate the coupling coordination degree and spatial connection between China's digital economy and sports industry. The coupling coordination degree between the two systems was observed to be relatively low. The findings also indicated substantial positive agglomerative effects, with the level of agglomeration rising each year. Li et al. (22) measured the coupling coordination of cultural, sports, and tourism industries across 31 provinces in China. The authors employed spatial autocorrelation methods to identify factors influencing their coordinated development from both global and local perspectives and to explore their underlying mechanisms. Scholarship on the sports industry has become increasingly rich and diverse. Using a range of methodologies, researchers have conducted in-depth analyses of its linkages with other industries, thereby laying a solid foundation for the present study.

The evolution of the sports industry and its growing integration with the health industry have prompted scholars to explore the relationship between the two industries. The current literature can be classified into two primary categories, namely, theoretical and quantitative research. Theoretical studies encompass the conceptual framework, historical context, driving factors, and implementation strategies for the integrated development of the two industries. Yang (23) argued that the aging population in China requires the integration of sports with care and health services for older adults, leading to substantial advancements in businesses related to leisure sports and senior care. Wang and Yin (24) conducted a systematic analysis of the driving factors for the integration of the two industries and proposed four related pathways. Wang et al. (25) conducted a literature review and inductive-deductive reasoning to explore the mechanisms, practical challenges, and development pathways for the integrated development of the health and sports industries. Lu et al. (26) conducted a literature review and expert interviews to define the concept of the integrated physical medicine industry. The authors identified five main service operation models currently present in the market: female health; physical fitness and health; physical education and medicine; chronic disease rehabilitation; and sports injury rehabilitation. Western scholars also recognize that investment in sport can generate public health and economic benefits. Kosnikova et al. (27) argue that participation in physical activity promotes health and wellbeing and that investing in sports infrastructure and broad-based participation programs can reduce healthcare expenditures, lower sickness absence, and increase labor productivity. Regions with higher sports participation rates tend to exhibit lower morbidity and higher economic efficiency. The second key research category, quantitative research, includes studies on the current state, challenges, and solutions regarding the integration of the two industries in a designated region. Liu (28) used econometric methods to validate the relationship between the two industries and their respective secondary sectors. Xu et al. (19) demonstrated that the complete development levels of the Chinese sports industry and health services exhibit a steady annual increase over the period 2013–2017. Zhuo et al. (29) developed an evaluative methodology for the integration of the sports and older adult care industries based on analysis of the coupling effect mechanism. The findings indicate that the synergistic interaction between the two industries increases each year. Li et al. (30) utilized the coupling coordination degree and standard deviation ellipse model to assess the coupling development level of the sports and medical integration industry in parallel with high-quality economic development in China, revealing a comparatively low coupling coordination level.

The current research findings have significant progress in the field, establishing a basis for this study. However, further investigation is still required in several areas. First, the connection between the health and sports industries is predominantly examined through qualitative research, with common methods including literature analysis and inductive–deductive reasoning. Conversely, research on quantitative evaluations lacks systematic rigor and depth. Second, existing research primarily concentrates on the coupling and cooperation between the sports and health industries, while studies investigating the direct impact of the sports industry on the development of the health industry are limited. Third, the health and sports industries are intricate systems. What is the impact of the sports industry on the health industry? Does it exert an amplifying or diminishing effect? What are the fundamental mechanisms? Are mediating or threshold effects present? These questions require further exploration.

This study addresses the aforementioned limitations in the current literature by using panel data from 30 provinces in China (excluding Hong Kong, Macau, Taiwan, and Tibet), spanning 2014 to 2023. We employed several econometric methods, including fixed-effects models, endogeneity tests, heterogeneity tests, robustness tests, mediation-effect tests, and threshold-effect tests, to investigate the mechanisms by which the sports industry affects the development of the health industry. Policy recommendations are subsequently developed based on the findings. This study is crucial for meeting individuals' expectations for improved quality of life, maximizing sports consumption potential, and providing vital insights to promote the formation of a Healthy China and regional coordinated development strategies.

## 3 Theoretical analysis and research hypothesis

### 3.1 Direct impact of the sports industry on the health industry

The sports industry centers on physical activities and enhances human health through the provision of sports-related products and services (31). The health industry seeks to sustain and improve health standards through medical care, health maintenance, and rehabilitation, thereby addressing the varied health requirements of the population (32). Moreover, the sports industry not only fulfills individuals' quest for a healthy lifestyle through a diverse range of athletic activities, coordinated events, extensive fitness services, and a broad spectrum of sports-related products (33), but also significantly propels the health industry forward (34). The advancement of the health industry depends on the public's heightened knowledge of health, as well as the increasing demand for health (35). The sports industry promotes public health consciousness and literacy by popularizing the notion of physical fitness and endorsing evidence-based exercise methods (36). There is a significant correlation between the sports and health industries in terms of service offerings and target demographics. The sports industry offers tailored exercise programs, comprehensive health assessment services, and other services that directly address the varied health management needs of individuals (37).

Consequently, the sports industry not only enhances health awareness across society but also directly stimulates the robust development of the health industry by providing professional health services and products (38).

Based on the above, we propose the following hypothesis,

H1: The sports industry can directly promote the development of the health industry.

### 3.2 Regional heterogeneity in the impact of the sports industry on the health industry

Regional heterogeneity produces differences in both the nature and intensity of the influence of the sports industry on the health industry across regions (10, 39). The relationship between the sports and health industries is not a one-dimensional linear interaction; rather, it is determined by numerous variables such as the regional economic development level, policy environment, and social culture. Consequently, the interaction between the two industries exhibits distinct regional heterogeneity.

In economically advanced regions, substantial consumer spending and well-developed service systems facilitate the integration of the sports and health industries. For example, in Beijing, the organization of major sporting events, such as the Beijing Winter Olympics, along with the promotion of national fitness initiatives, not only raises public health awareness but also accelerates the growth of related sectors, including sports equipment and health monitoring devices (40).

Variations in the policy environment are a key factor contributing to regional heterogeneity. In particular, regional disparities in governmental support, resource allocation, and industrial planning for the sports and health industries directly affect their interaction (41). Zhejiang Province in eastern China has advanced the collaborative development of the sports and health industries by implementing the “Healthy Zhejiang” Action Plan. This initiative explicitly promotes the advancement of sports rehabilitation equipment and health monitoring technologies, thereby fostering strong cooperation between the sports and health industries (42, 43). Conversely, regions with minimal policy support exhibit relatively constrained synergistic effects between the two industries.

Socio-cultural factors play a significant role in influencing regional heterogeneity. Variations in health knowledge, exercise practices, and consumer behaviors across regions further affect the ability of the sports industry to promote the development of the health industry. In Northern European countries such as Norway and Sweden, heightened health awareness and active participation in sports have led to a strong preference for outdoor activities and exercise routines (44). This cultural trait has driven the rapid growth of local sports equipment and health service sectors.

Based on the above, we propose the following hypothesis,

H2: The impact of the sports industry on the health industry exhibits regional heterogeneity.

### 3.3 Indirect impact of the sports industry on the health industry

R&D intensity serves as a crucial metric for assessing industrial innovation capabilities and technological progress (45). As a field closely linked to health needs, the sports industry's increased R&D intensity not only drives innovation and improvement in sports products and services but also directly promotes the development of the health industry through technological spillover effects and knowledge diffusion mechanisms (46). R&D activities in the sports industry primarily focus on sports equipment, smart wearable devices, sports rehabilitation technologies, and sports facilities (47, 48). Significant technical support and service assurances for the health industry are provided by product improvements and technological breakthroughs in these areas (49). The creation of intelligent wearable technology, for instance, enables real-time monitoring and analysis of human health data, providing a scientific basis for health management (48, 50, 51). Technological advancements in sports rehabilitation enhance the prevention and management of sports injuries, thereby reducing the likelihood of health problems associated with sports participation (52).

Based on the above, we propose the following hypothesis,

H3: R&D intensity mediates the relationship between the sports industry and the health industries.

### 3.4 Threshold effect of the development level of the sports industry

The positive impact of the sports industry on the health sector does not appear immediately; it develops gradually and becomes stable only after the sector attains sufficient scale and quality. In the initial stage, incomplete infrastructure, an inadequate supply of sports services, and limited public health awareness constrain the marginal contribution of the sports industry (53). Payment and regulatory barriers, such as the incomplete inclusion of exercise prescription, rehabilitation, and health management programs in public reimbursement schedules and commercial benefit packages—together with shortfalls in human resources and data systems, including shortages of sports medicine and rehabilitation professionals and weak interoperability between electronic medical records and wearable device data, hinder the conversion of sports-related spillovers into effective demand within the health industry. Practical constraints, including a limited stock of sports facilities, low participation rates, and insufficient professionalization of health services, further dampen the positive spillover. Only when the sports industry crosses critical thresholds in coverage and professionalization does its capacity to promote the health industry increase markedly.

As the sports industry continues to expand, the marginal contribution of additional investment declines. On the infrastructure side, once facility coverage reaches saturation, merely increasing supply does not meaningfully raise participation in physical activity or improve population health. On the demand side, as physical fitness improves and the risk of chronic disease declines, some curative services are replaced by preventive and management services, which dilutes the net demand-pull effect on the health industry.

Regarding the matching of supply and demand, when the supply structure of the sports industry does not align with the heterogeneous health needs of different populations, the marginal benefits of additional resources decline further. Without targeted, stratified service provision by age, sex, and health status—for example, personalized bundles of rehabilitation, nutrition, exercise interventions, and health management—the utilization of new resources is unlikely to improve. Moreover, the sports industry influences the health sector through multiple channels, including sports consumption, sporting events, nationwide fitness initiatives, and health education; however, the synergies among these channels are not simply additive. Once development reaches a certain level, the scope for further gains in coordination efficiency becomes limited, producing an overall pattern of diminishing marginal effects with a non-linear profile.

Based on the above, we propose the following hypothesis,

H4: The impact of the sports industry on the health industry exhibits a threshold effect and non-linear diminishing marginal returns.

**Figure 1:**
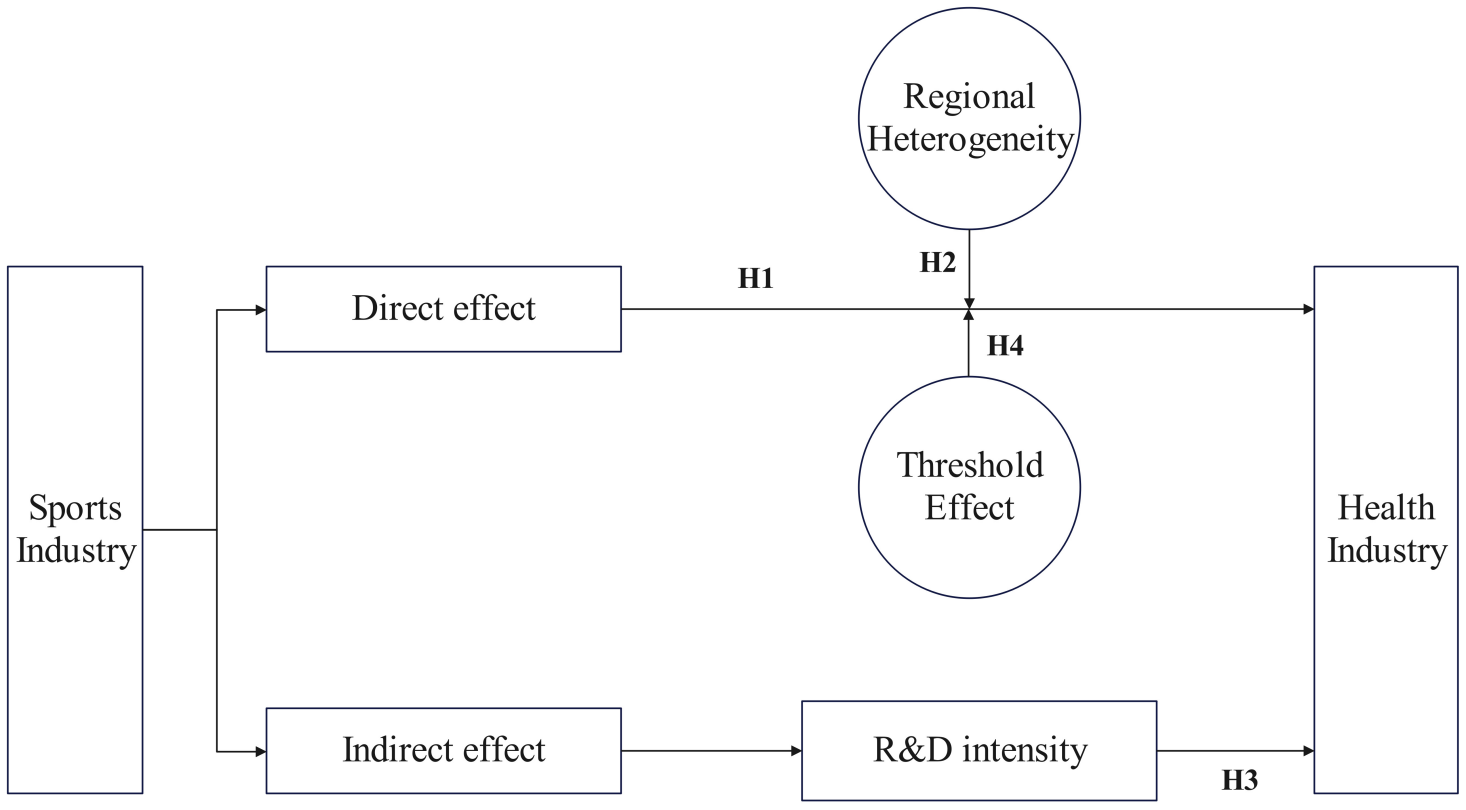
Theoretical model.

Accordingly, a theoretical model is constructed to elucidate the mechanisms by which the sports industry promotes the development of the health industry, as shown in Figure 1.

## 4 Methods and data

### 4.1 Definition of variables

#### 4.1.1 Dependent variable

The health industry (HID) evaluation indicators are drawn from previous literature, including Xu et al. (19), Shu et al. (54), and Hu et al. (55), with a focus on four dimensions (Table 1): medical insurance, pharmaceutical industry, health management, and health environment to assess the health industry development level. Medical insurance refers to the quantity and standardization of primary healthcare services. The pharmaceutical industry, as a fundamental element of the national health industry (15), includes indicators such as the output value of the pharmaceutical manufacturing sector and the number of enterprises, reflecting the overall benefits of the biopharmaceutical industry in different regions. Health management pertains to the accessibility and security of citizens in obtaining healthcare, as well as their expectations in this context. The health environment refers to the carrying and ecological capacities of regional habitats, with the ecological environment reflecting the quality of the living environment for public health. All 12 indicators reported in Table 1 are positive, and HID is calculated using the entropy method.

**Table 1:** Health industry evaluation indicators and weights.

**Target**	**Dimensions**	**Indicators**	**Units**	**Symbols**	**Weights**
Health industry	Medical insurance	Number of medical and health institutions	Individual	H1	0.0891
		Number of health workers	10,000 people	H2	0.0680
		Local government expenditure on healthcare	100 million Yuan	H3	0.0650
		Number of participants in urban basic medical insurance at the end of the year	10,000 people	H4	0.0879
	Pharmaceutical industry	Number of legal entities in the pharmaceutical manufacturing industry (number of industrial enterprises above the designated size)	Individual	H5	0.0946
		Pharmaceutical manufacturing output value	100 million Yuan	H6	0.1199
	Health management	Number of outpatients for health examination at medical and health institutions	10,000 people	H7	0.1006
		Number of community health service centers	Individual	H8	0.1047
		Number of legal entities in public administration, social security, and social organizations	Individual	H9	0.0712
	Health environment	Forest coverage	%	H10	0.0618
		Green coverage ratio of the built-up area	%	H11	0.0155
	Completed investment in industrial pollution Control	10,000 Yuan	H12	0.1217

#### 4.1.2 Independent variable

Based on research by Ren (56, 57), Kang and Huang (58), Wang et al. (21), and Cheng et al. (20), the development level of the sports industry (SID) is assessed through three dimensions: industrial dynamics, industrial efficiency, and market scale. Industrial dynamics act as the essential foundation and impetus for the advancement of the sports business. Industrial efficiency reflects the outcomes and advantages produced by the sports industry's advanced development, highlighting the inventive potential of its manufacturing processes. Market scale refers to the number of market participants and workforce involved in the sports business, serving as an indicator of its market vitality. Given the sports industry's recent emergence, its statistical data remain underdeveloped and limited. Wang et al. (21) and Su (59) connected to the culture, sports, and entertainment industries, are selected as the sports industry indices (Table 2). All indicators are positive, and SID is calculated using the entropy method.

**Table 2:** Sports industry evaluation indicators and weights.

**Target**	**Dimensions**	**Indicators**	**Units**	**Symbols**	**Weights**
Sport industry	Industrial efficiency	Government expenditure on culture, sports, and entertainment	100 million Yuan	S1	0.0879
		Per capita area of sports facilities	m2	S2	0.0471
		Investment in fixed assets within the cultural, sports, and entertainment sectors	100 million Yuan	S3	0.1657
	Industrial efficiency	Sports lottery sales	100 million Yuan	S4	0.1288
		Total wage of urban employed personnel in the cultural, sports, and entertainment sectors	100 million Yuan	S5	0.2241
		Average wage of urban private sector employees in the cultural, sports, and entertainment industries	Yuan	S6	0.0735
	Market scale	Number of legal entities in the cultural, sports, and entertainment sectors	Individual	S7	0.1636
		Number of employed personnel in urban units of the cultural, sports, and entertainment industries	10,000 people	S8	0.1093

#### 4.1.3 Mediator variable

Building on previously reported theoretical analyses (16, 41), this study posits that SID enhances HID by increasing R&D intensity. Here, R&D intensity is defined as the ratio of internal R&D expenditure to regional GDP.

#### 4.1.4 Control variables

The following six control variables are selected from previous studies (41, 60, 61) to evaluate the effect of SID on HID and to minimize the influence of extraneous factors: economic development level (lnPGDP); urbanization level (URB); degree of external openness (OPE); industrial structure upgrading (IUP); human resource support (HUM); and aging rate (AGE). Table 3 presents the variable definitions.

**Table 3:** Definition of all variables.

**Variable type**	**Abbreviation**	**Variable name**	**Definition**
Dependent variable	HID	Health industry	Measurement of the development level of the health industry
Independent variable	SID	Sports industry	Measurement of the sports industry development level
Mediator variable	RD	R&D Intensity	Internal expenditure on R&D/Regional GDP
Control variables	LnPGDP	Economic development level	Logarithmic GDP per capita
	URB	Urbanization level	Number of resident populations in towns/number of resident populations in districts
	OPE	Degree of external openness	(Total exports and imports of goods ^*^ US dollar to RMB exchange rate)/GDP
	IUP	Industrial structure upgrading	Tertiary sector output/secondary sector output
	HUM	Human resource support	Students enrolled in general higher education institutions/the entire regional population
	AGE	Aging rate	Population over 65 years of age/total regional population

#### 4.1.5 Descriptive statistics of variables

As reported in Table 4, the development of a health industry (HID) exhibits marked variation, with an average of 0.259, a maximum of 0.690, and a minimum of 0.0158. Similarly, the development of the sports industry (SID) also presents marked variation. The control variable values, including lnPGDP (mean of 9.338), URB (mean of 0.625), and AGE (mean of 12.22), are within acceptable limits. The variables across the 30 selected provincial units display notable discrepancies, establishing a basis for the empirical study of the potential influences and non-linear spillover effects of the sports industry on the health industry.

**Table 4:** Descriptive statistics.

**Variables**	**Obs**	**Mean**	**Std. Dev**.	**Min**	**Max**
HID	300	0.259	0.154	0.0158	0.690
SID	300	0.181	0.124	0.0247	0.697
RD	300	0.0186	0.0119	0.00446	0.0683
LnPGDP	300	9.338	0.468	8.660	10.81
URB	300	0.625	0.109	0.402	0.894
OPE	300	0.252	0.243	0.00763	1.134
IUP	300	1.469	0.781	0.704	5.690
HUM	300	0.0225	0.00583	0.00918	0.0437
AGE	300	12.22	3.042	6.778	21.06

### 4.2 Model construction

#### 4.2.1 Baseline regression model

This study constructs the following baseline regression model:


$$\begin{array}{c} HID_{it}=a_{0}+a_{1}SID_{it}+\varepsilon_{it} \end{array}$$ (1)

Equation 1 may exhibit estimation errors, and thus, we incorporate control variables to derive the subsequent two-way fixed effects model:


$$\begin{array}{c} HID_{it}=a_{0}+a_{1}SID_{it}+{a_{n}C}_{it}+\mu_{it}+\gamma_{it}+\varepsilon_{it} \end{array}$$ (2)

Where *i* represents the province; *t* denotes the year; ε_*it*_ is a random disturbance term. *HID*_*it*_ is the dependent variable; *SID*_*it*_ is the independent variable; *C*_*it*_ denotes the control variables; μ_*it*_ represents the individual fixed effects; γ_*it*_ denotes the time fixed effects; *a*_0_ is the constant term; and *a*_1_ and *a*_*n*_ are the regression coefficients.

#### 4.2.2 Mediation effect model

Building on the approach of Jiang (62), this study employs a two-step method to construct a mediation effect model and to validate the mediating role of R&D intensity. Regression analysis was initially conducted with the sports industry as the independent variable and the health industry as the dependent variable (Equation 1). Following this, R&D intensity was set as the dependent variable, with the sports industry as an independent variable (Equation 3). The findings were then integrated with previous studies to evaluate the impact of R&D intensity on the health industry.


$$\begin{array}{c} RD_{it}=\beta_{0}+\beta_{1}SID_{it}+{\beta_{n}C}_{it}+\mu_{it}+\gamma_{it}+\varepsilon_{it} \end{array}$$ (3)

Where *i* represents the province; *t* denotes the year; ε_*it*_ is a random disturbance term; *RD*_*it*_ is the dependent variable; *SID*_*it*_ is the independent variable; *C*_*it*_ represents the control variables; μ_*it*_ accounts for the individual fixed effects; γ_*it*_ represents the time fixed effects; β_0_ is a constant term; β_1_ and β_*n*_ are the regression coefficients.

#### 4.2.3 Threshold regression model

Equation 4 describes a threshold model with *SID* as the threshold variable.


$$\begin{array}{l} HID_{it}=\phi_{0}+\phi_{1}SID_{it}\times \;I(HID_{it}\leq \theta )+\phi_{2}HID_{it}\times \;I(HID_{it}\theta )\\ \;+\;\phi_{n}C_{it}+\mu_{it}+\gamma_{it}+\varepsilon_{it} \end{array}$$ (4)

where *I*(·) represents the indicative function, with a value of 0 when the expression in parentheses is false, and 0 otherwise; θ is the threshold value; ϕ_0_ is the constant term; ϕ_1_, ϕ_2_ and ϕ_*n*_ denote the regression coefficients.

### 4.3 Data sources

In 2014, China released the “Opinions on Accelerating the Development of the Sports Industry and Promoting Sports Consumption,” which explicitly called for “Foster the mutual integration between the sports industry and other industries” and “Enrich the sports industry's content; promote its integration with older adult care services, the cultural and creative industries and design services, and education and training; and stimulate the development of related subsectors, including sports tourism, sports media, sports conventions and exhibitions, sports advertising, and sports film and television.” Following this strategy, a series of programmatic documents was issued to promote the integration and enhancement of sports and health, contributing to the expansion of the health industry and ushering the sports industry into a new phase of development. This study utilizes panel data spanning 2014–2023 from China's 30 provinces (excluding Hong Kong, Macau, Taiwan, and Tibet) within the health and sports industries. The data were sourced from the China Statistical Yearbook and provincial statistical yearbooks. Missing data points were addressed using linear interpolation. The original data of the selected indicators were normalized using the range standardization method.

## 5 Estimation and result

### 5.1 Baseline regression result

A fixed-effects model was selected to assess the impact of the sports industry on the health industry based on the Hausman test results. In Column (1) of Table 5, without controlling for province, year, or control variables, the regression coefficient of SID is 0.886, which is statistically significant at *p* = 0.01. In Column (2), after controlling for province and year, the regression coefficient for the sports industry on the health industry increases to 0.962, which is also statistically significant at the *p* = 0.01 level. Compared to Column (1), the regression coefficient in Column (2) increases, indicating the potential presence of omitted variable bias in Column (1). After controlling for province and year, the positive effect of the sports industry on the health industry is amplified. When the control variables are included, the regression model's coefficient for the relationship between the sports and health industries rises to 1.001 (Column 3). This result remains statistically significant at *p* = 0.01. This finding supports H1 by indicating that SID effectively promotes the development of HID.

**Table 5:** Baseline regression results.

**Variables**	**HID**		
	**(1)**	**(2)**	**(3)**
SID	0.886^***^ (0.0034)	0.962^***^ (0.0000)	1.001^***^ (0.0000)
LnPGDP			0.1330^***^ (0.0001)
URB			−0.1475 (0.3033)
OPE			−0.2186^***^ (0.0001)
IUP			0.0374^***^ (0.0000)
HUM			−10.0866^***^ (0.0000)
AGE			0.0104^***^ (0.0000)
Constant	0.0986^**^ (0.0228)	0.0847^***^ (0.0000)	−0.9727^***^ (0.0003)
Control variables	NO	NO	Yes
Individual effect	NO	Yes	Yes
Time effect	NO	Yes	Yes
Observations	300	300	300
adj. *R*^2^	0.5018	0.5082	0.6527

*p*-values in parentheses; ^**^p < 0.05, ^***^p < 0.01.

### 5.2 Robustness tests

#### 5.2.1 Winsorization test

This study re-conducted the regression analysis after applying a 1% winsorization to all variables in the baseline regression to reduce the impact of extreme variable values on the regression results. SID continues to have a beneficial effect on HID, as evidenced by the sports industry's coefficient of 1.022 (Table 6), which is statistically significant at the *p* = 0.01 level. This outcome remains consistent with the baseline regression results.

**Table 6:** Robustness tests using winsorization.

**Winsorization test**	
**Variables**	**HID**
SID	1.022^***^ (0.0472)
Constant	−1.048^***^ (0.260)
Control variables	YES
Individual effect	YES
Time effect	YES
Observations	300
Number of years	10
*R* ^2^	0.688

Standard errors in parentheses; ^***^p < 0.01.

#### 5.2.2 Partial sample exclusion

Four Chinese municipalities (Beijing, Shanghai, Tianjin, and Chongqing) were excluded from the analysis. Column (1) of Table 7 reveals the sports industry regression coefficient to be 1.375, which is statistically significant at *p* = 0.01. This indicates that SID persistently contributes positively to HID.

**Table 7:** Robustness tests after partial sample exclusion.

**Variables**	**HID**	
	**Exclude Beijing, Shanghai, Tianjin, and Chongqing**	**Exclude 2020–2022**
	**(1)**	**(2)**
SID	1.375^***^ (0.0407)	1.061^***^ (0.0649)
LnPGDP	0.107^***^ (0.0254)	0.119^***^ (0.0410)
URB	−0.246^**^ (0.103)	−0.0973 (0.181)
OPE	−0.0731^*^ (0.0388)	−0.200^***^ (0.0650)
IUP	0.0118^*^ (0.00644)	0.0392^***^ (0.0108)
HUM	3.644^***^ (1.309)	−10.03^***^ (1.698)
AGE	−0.00138 (0.00194)	0.0103^***^ (0.00327)
Constant	−0.873^***^ (0.202)	−0.883^***^ (0.328)
Individual effect	YES	
Time effect	YES	
Observations	260	210
Number of years	10	7
*R* ^2^	0.853	0.643

Standard errors in parentheses; ^***^p < 0.01, ^**^p < 0.05, ^*^p < 0.1.

The analysis was modified by eliminating the COVID-19 pandemic years (2020–2022) to remove its impact on the pandemic. Compared to the model that included pandemic years (1.001), the regression coefficient rises to 1.061, as shown in Column (2) of Table 7, and the significance level remains unchanged. Overall, the findings suggest that the benefits of the sports industry for the health industry continued throughout the pandemic (63). This is explained by the pandemic's increased emphasis on sanitation and healthcare (64), which opened up new business prospects for the health industry. The pandemic spurred the adoption of online sports and fitness activities. This led to a surge in public demand for sports and an increased awareness of the importance of physical health through sports activities (65, 66).

#### 5.2.3 Inclusion or reduction of control variables

Two additional specifications were implemented to assess the results' robustness: (1) removing the control variables HUM and URB; and (2) adding new control variables, including government intervention, fiscal support intensity, and aging consumption demand. In both specifications, the regressions were statistically significant at the 1% level (Table 8), demonstrating the strong robustness of the empirical results.

**Table 8:** Robustness tests after excluding and including control variables.

**Variables**	**HID**	
	**Exclude control variables**	**Include control variables**
	**(1)**	**(2)**
SID	0.922^***^ (0.0525)	0.971^***^ (0.0489)
LnPGDP	0.113^***^ (0.0305)	0.0854^**^ (0.0365)
URB		0.0504 (0.167)
OPE	−0.251^***^ (0.0567)	−0.280^***^ (0.0660)
IUP	0.0305^***^ (0.00960)	0.0544^***^ (0.00993)
HUM		−11.32^***^ (1.592)
AGE	0.00113 (0.00255)	0.00702^**^ (0.00292)
Aging consumption demand		−0.459 (0.479)
Fiscal support intensity		−0.931^**^ (0.457)
Government intervention		0.692 (0.469)
Constant	−0.956^***^ (0.273)	−0.488 (0.304)
Individual effect	YES	
Time effect	YES	
Observations	300	300
Number of years	10	10
*R* ^2^	0.574	0.690

Standard errors in parentheses; ^***^p < 0.01, ^**^p < 0.05.

### 5.3 Endogeneity treatment

Reverse causality between the dependent and independent variables can lead to endogeneity. In particular, the health and wellness industry in a region may also promote sports industry development, potentially leading to biased regression results. To mitigate this potential endogeneity concern, this study employs a Two-Stage Least Squares approach (2SLS), based on previous research (57), treating SID as an endogenous variable. A lagged value of SID (lagged by one period) is utilized as the instrumental variable. Since the lagged sports industry is not directly affected by the current period's shocks and is uncorrelated with the error term, this methodology alleviates the endogeneity issue arising from reverse causality, providing a more robust estimation of the baseline model.

Table 9 reports the estimation results. In the first-stage regression, a significant positive correlation is observed between SID and the instrumental variable. In the second-stage regression, the Kleibergen–Paap rk LM statistic is statistically significant at the 1% level, and the Kleibergen–Paap rk Wald *F* statistic is 1,130.093. The minimum eigenvalue statistic exceeds the 10% critical value (16.38), rejecting the null hypothesis and indicating that the lagged SID is not a weak instrument. Thus, the selected instrumental variable is valid. After addressing potential endogeneity issues using the instrumental variable, the sports industry's positive impact on the health industry remains statistically significant, consistent with baseline regression results. This suggests that SID continues to effectively promote HID.

**Table 9:** 2SLS regression results.

**Variables**	**First stage**	**Second stage**
	**Lag_SID-SID**	**SID-HID**
	**(1)**	**(2)**
Lag_SID	0.961^***^ (0.029)	
SID		0.186^***^ (0.059)
Constant	−0.202 (0.125)	−0.352^**^ (0.156)
Control variables	YES	
Individual effect	YES	
Time effect	YES	
Kleibergen–Paap rk LM statistic	14.099^***^	
Kleibergen–Paap rk Wald *F* statistic	1,130.093 [16.38]	
Observations	270	270
*R* ^2^	0.932	0.527
F	399.31	26.262

Standard errors in parentheses; critical values for the Stock-Yogo weak identification test at the 10% level are in square brackets; ^**^p < 0.05, ^***^p < 0.01.

### 5.4 Heterogeneity analysis

#### 5.4.1 Regional heterogeneity

The developmental status of SID varies among regions, and its effect on HID exhibits notable heterogeneity. Consequently, the 30 provinces in the study sample were classified into four regions: namely, eastern, central, western, and northeastern, and regression estimation was performed independently on each region (Table 10).

**Table 10:** Regional heterogeneity test results.

**Variables**	**HID**				
	**(1)**	**(2)**	**(3)**	**(4)**	**(5)**
	**10%**	**25%**	**50%**	**75%**	**90%**
SID	0.268^***^ (4.83)	0.263^***^ (6.07)	0.254^***^ (6.78)	0.245^***^ (4.12)	0.238^**^ (2.90)
Control variables	YES				
Individual effect	YES				
Time effect	YES				
Observations	300				

*p*-values in parentheses; ^**^p < 0.05, ^***^p < 0.01.

The positive impact of SID on the HID is statistically significant (at the 1% level) in the eastern, central, and western regions. The magnitude of the impact for each region, from lowest to highest, is as follows: central (0.4787) <east (0.9904) <west (1.7361). Conversely, SID does not exert a statistically significant influence on HID in the northeastern region. This reveals considerable regional variability.

Potential explanations for this variability include the advanced economy in the eastern region, the diversified industrial structure, the relatively minor contribution of the sports industry to the overall economy, and the more mature development of the health industry compared to the sports industry, which may diminish the direct influence of the latter on the former. In the comparatively underdeveloped economy of the western region, the sports industry may play a crucial role in advancing the health sector, driven by targeted policies and focused resource investment, resulting in a more pronounced impact on health. Compared with the eastern and western regions, SID in the central region may also significantly contribute to HID. However, during the economic transition period, supporting policies and resource allocation are relatively limited, with a relatively weak impact, although they continue to play a certain promotional role. The northeastern region of China, as an aging industrial hub, faces several constraints, including sluggish industrial transformation, brain drain, and the decline of a resource-dependent economy. Consequently, the sports industry is relatively underdeveloped, leading to a diminished impact of the SID on HID. As an old industrial base, the northeastern region has experienced slow industrial restructuring, population outflows, and a downturn in resource-dependent economies, which together impose multiple constraints on the capacity of the sports industry to stimulate the health industry. Several mechanisms may account for this pattern. First, there are demand and payment constraints: sluggish growth in per capita disposable income, net out-migration of younger cohorts, and pronounced population aging weaken willingness to spend on sports consumption and preventive health care, making it difficult to translate sports spillover effects into effective demand for health services. Second, the quality and structure of sports supply are insufficient: facilities are outdated, spatial layout and accessibility are suboptimal, the supply of high-value-added services such as events and training camps is limited, professional capabilities in sports medicine, rehabilitation, and health management are inadequate, and the complementary chain linking sports to health remains short. Third, human capital and digital foundations are weak, with net outflows of rehabilitation therapists, sports medicine physicians, and health managers, and limited interoperability between electronic medical records and data from wearable devices. Fourth, climate and operating costs matter: long, cold winters raise travel and operating costs; outdoor activities are highly seasonal; and energy use in indoor venues is high, all of which reduce the efficiency with which incremental sports supply is converted into health output. Fifth, homogenization and early saturation are evident: winter sports deployments are relatively concentrated, but product portfolios are homogeneous, competition across localities tends to be zero-sum, and additional supply has limited leverage on incremental health demand. The empirical results support H2.

#### 5.4.2 Quantile regression model estimation

To examine the variability in the effect of SID across different levels of HID, we performed a quantile regression analysis in accordance with the literature (67). We selected the 10th, 25th, 50th, 75th, and 90th percentiles to analyze the heterogeneous effects of the sports industry on the health industry (Table 11).

**Table 11:** Quantile regression model estimation results.

**Variables**	**HID**				
	**(1)**	**(2)**	**(3)**	**(4)**	**(5)**
	**10%**	**25%**	**50%**	**75%**	**90%**
SID	0.268^***^ (4.83)	0.263^***^ (6.07)	0.254^***^ (6.78)	0.245^***^ (4.12)	0.238^**^ (2.90)
Control variables	YES				
Individual effect	YES				
Time effect	YES				
Observations	300				

T statistics in parentheses; ^**^p < 0.01, ^***^p < 0.001.

The regression results in Table 11 indicate that the effect of SID on HID is statistically significant at or above the 1% level across the five quantile points. Regression coefficients vary across quantiles, declining as the quantile increases. This indicates that the impact of the sports industry on the health industry varies with its level of growth. Specifically, although SID consistently demonstrates a positive connection with HID, the relationship is non-linear and fluctuates across several developmental phases. This suggests that at lower tiers of health industry development (10 and 25%), the sports industry demonstrates the most pronounced positive impact on the health industry. This pattern may reflect conditions at the early stage of health industry development, when medical resources, technological infrastructure, and specialized personnel are scarce. Physical activity initiatives, such as community fitness facilities and nationwide fitness programs, offer advantages of low investment, rapid diffusion, and low barriers to participation, allowing swift coverage of large populations. Thus, the sports industry becomes an important vehicle for promoting health, and the concentration of policy support and resource inputs produces a clear and measurable impetus. As HID rises (50 and 75%), the promotional effect of the sports industry remains significant, but with a noticeable decline in its impact intensity. At higher levels of HID (90%), the impact remains statistically significant at the 1% level, though its magnitude is smaller. This pattern may arise at an advanced stage of health industry development, when the value chain has become relatively mature and spans multiple segments, including medical care, rehabilitation, health management, and intelligent devices. As one component within this expanded system, the relative influence of the sports industry is diluted by other segments and no longer constitutes the principal driver of sectoral growth. Moreover, as development levels increase, medical technology, precision medicine, and artificial intelligence-assisted diagnosis play an increasingly important role in the provision of health services. Compared with physical activity and other prevention-oriented and lifestyle interventions, technology-driven medical services tend to deliver more direct and substantial improvements in health outcomes.

### 5.5 Mediating effect tests

The analysis in Section 5.4 validates the influence of the sports industry on the health industry and withstands robustness checks, supporting a reliable research conclusion. Our theoretical analysis suggests that the sports industry may indirectly influence the development of the health industry by affecting R&D intensity. Following the approach of Jiang (62), we employed a two-step mediation effect model to examine the mechanism of action. Since the causal relationship between the mediator variable (RD) and the dependent variable (HID) is theoretically intuitive, in the mediation effect test, we focus just on the relationship between the independent variable (SID) and the mediator variable (RD).

Table 12 reports the results of the mediation effect. Column (1) in Table 12 and the previously discussed baseline regression results (Table 5) demonstrate that SID can effectively enhance HID, yet the underlying mechanism requires further verification. Column (2) of Table 12 reveals that the coefficient of SID is 0.0146. This is statistically significant at the 1% level and indicates the positive influence of the sports industry on RD. Current research suggests that the health industry, particularly the production of medical supplies, is fundamentally reliant on R&D, which necessitates substantial investments (68). Moreover, artificial intelligence technology has markedly improved urban public health resilience by optimizing resource allocation and operational efficiency (69). Similarly, R&D has significantly enhanced management efficiency, accelerated emergency response, and improved resource allocation in public healthcare (70). Banerji's empirical analysis shows a positive association between R&D intensity, patent counts, the number of regulatory applications, and the export intensity of Indian pharmaceutical firms. It indicates that R&D investment significantly enhances firms' export capacity. Kuittinen et al. (71) found that R&D intensity is significantly higher in science-based industries, such as health care, and is positively related to firm performance. By extension, innovation and research in the sports industry can drive health industry development. In practice, innovation in the sports sector through digital technologies, wearable devices, and rehabilitation applications can strengthen its linkage with health services. For example, intelligent wearables can monitor activity data in real time and provide personalized training guidance, improving exercise effectiveness and safety; the integration of large-scale sports data with artificial intelligence facilitates early detection of health risks and supports chronic disease management. Furthermore, rehabilitation applications grounded in exercise science are advancing the intelligent and home-based delivery of services for post-operative recovery and functional restoration among older adults. These technological advances not only expand the boundaries of the sports industry but also promote greater precision and personalization in health services, enabling a shift from reactive medical care to proactive health. These conclusions are consistent with the evidence in this study and support hypothesis H3.

**Table 12:** Mediating effect tests.

**Variables**	**HID**	**RD**
	**(1)**	**(2)**
SID	1.001^***^ (0.0000)	0.0146^***^ (0.0070)
Constant	−0.9727^***^ (0.0003)	0.0310^*^ (0.0811)
Control variables	YES	
Individual effect	YES	
Time effect	YES	
Observations	300	300
adj. *R*^2^	0.6527	0.9867

*p*-values in parentheses; ^*^p < 0.1, ^***^p < 0.01.

### 5.6 Threshold regression analysis

Before conducting empirical analysis with a threshold model, we tested whether a threshold effect exists when SID is treated as a threshold variable. Table 13 reports the results. The single threshold meets the statistical significance criterion at 10%, suggesting that when SID is treated as a threshold variable, the model demonstrates a threshold effect. The presence of a threshold effect indicates that threshold analysis should be conducted. The double threshold is significant at the 5% level, while the triple threshold fails to meet the criterion. Consequently, when SID is regarded as a threshold variable, a double-threshold effect arises between SID and HID.

**Table 13:** Threshold effect tests.

**Threshold Variable**	**Threshold type**	**Fstat**	***p* Value**	**Critical value**			**Repeated sampling times**
				**10%**	**5%**	**1%**	
SID	Single	57.49	0.0000	40.9379	43.5424	46.7178	300
	Double	13.57	0.0400	10.7899	12.7572	16.7796	
	Triple	7.76	0.8933	23.1711	25.3539	27.7595	

Table 14 presents the results of the double threshold model for two threshold values. When SID is at a low level and drops below the first threshold value (SID ≤ 0.3225), it demonstrates a significant beneficial influence on HID, with the maximum effect (φ = 1.371). This implies that, when SID ≤ 0.3225, each one-unit increase in SID corresponds to a 1.371-unit increase in HID. This may be attributed to underdeveloped infrastructure and policy support for the sports industry during the early stages of development. Consequently, each additional investment yields substantial marginal advantages, thereby exerting a huge promotional impact on HID. At the early stage of development, a scarcity-and-catch-up effect predominates. Infrastructure, service provision, and institutional support are still nascent, so any additional investment in the sports industry can substantially relieve bottlenecks in venues, human resources, and delivery channels. Such investment generates strong positive spillover benefits for the health industry, including medical rehabilitation, health management, sports rehabilitation equipment, and digital health, thereby significantly promoting the development of the health industry.

**Table 14:** Regression results for the threshold effect.

**Variables**	**Regression result**
SID (SID ≤ 0.3225)	1.371^***^ (0.139)
SID (0.3225 <SID ≤ 0.4968)	1.143^***^ (0.0294)
SID (SID > 0.4968)	0.639^***^ (0.0685)
Constant	−0.657^***^ (0.142)
Control variables	YES
Individual effect	YES
Time effect	YES
Observations	300
*R* ^2^	0.743

Robust standard errors in parentheses; ^***^p < 0.01.

When the SID development index is between the range of the first and second threshold values (0.3225 <SID ≤ 0.4968), the sports industry continues to exert a considerable positive influence on HID, but with a diminished effect (φ = 1.143). Within this interval, coordination constraints emerge. Synergy between the sports and health sectors requires institutional complements, including harmonized standards, reimbursement mechanisms, and interoperable data systems. In their absence, factor misallocation may arise (e.g., capital may be directed to events and venues rather than to rehabilitation and service provision), and the supply of skilled personnel and appropriate technologies may lag. Although the net effect remains positive, it begins to display a diminishing trend.

As SID development increases above the second threshold value (SID > 0.4968), the positive impact of the sports industry on the health industry diminishes further (φ = 0.639). At this stage, crowding out and substitution effects become prominent. The marginal efficiency of capital, land, and fiscal subsidies declines, while redundant construction and homogeneous competition intensify. On the demand side, spending on sports entertainment may substitute for health-related consumption, and resources may concentrate in competitive and performance sports, which weakens the coupling with the health industry. Consequently, the capacity of the sports industry to promote the health industry diminishes further.

Accordingly, at the initial stage of development, the sports industry exerts an expansionary effect on the health industry. Nevertheless, the promotive impact does not increase monotonically with further improvements in the sports industry's development level, indicating a threshold range within which the effect operates. The empirical findings corroborate hypothesis H4.

## 6 Conclusions

This study utilized panel data from 30 provinces in China spanning 2014 to 2023. Baseline regression, mediation analysis, and threshold regression were conducted to investigate the impact of the sports industry on the development of the health industry. Based on the results, we determined the following conclusions.

The baseline regression findings demonstrated that the sports industry substantially fosters the development of the health industry. This conclusion remained robust after multiple robustness tests and endogeneity examinations, including winsorizing the variables, altering the sample period, and adjusting control variables.

The heterogeneity study indicated that the influence of the sports industry on the development of the health industry varied by region. The effect was more pronounced in western regions compared to eastern regions and more pronounced in eastern regions than in central regions. However, the impact in the northeastern region was not statistically significant. Furthermore, the influence of the sports industry on the health industry was most pronounced at the lower quantile level.

Mediating mechanism tests suggest that the sports industry can drive the health industry through R&D intensity.

The threshold test results demonstrated that the sports industry had a double threshold effect on the health industry, characterized by a non-linear pattern of diminishing marginal effects. When the development level of the sports industry was comparatively low, it greatly enhanced the advancement of the health industry. As the development level increased into the first and second threshold values, the promotional impact remained positive but weakened; even above the second threshold value, the marginal effect flattened rather than disappearing, and it remained statistically significant. Accordingly, the overall conclusion that the sports industry promotes health-industry development still holds; what changes is the magnitude of the marginal contribution, which progressively declines with higher development levels.

## 7 Theoretical and practical implications

This study highlights the pivotal role of the sports industry in advancing health industry development, offering vital data for policymakers seeking to enhance preparedness and response strategies. First, to enhance the synchronization of sports and health, it is imperative to utilize the directive function of policy planning. It is essential to encourage collaboration between medical institutions and sports organizations and advocate for “exercise prescriptions” and sports rehabilitation services. Furthermore, assistance should be provided for the integration of sports facilities, events, and health services, ultimately creating a policy support framework for “sports + health.” Second, market-driven innovation significantly contributes to the advancement of the health industry through the sports industry. The commercialization of the sports industry can enhance research and the promotion of health-related products. The amalgamation of intelligent wearable devices with health monitoring technology satisfies the requirements of fitness enthusiasts while also facilitating health data analysis. Moreover, the integration of sporting events with health-related consumption, including health tourism and nutritious food, has the potential to markedly broaden the health industry. Third, it is essential to enhance the comprehensive integration of the industrial chain to attain the synchronized advancement of the sports and health industries. The amalgamation of sports equipment and health technology has produced intelligent health devices, while the convergence of sports events and health services has resulted in initiatives such as sports injury rehabilitation and health consultation. Fourth, although the sports industry has the capacity to substantially improve the growth of the health industry, it demonstrates considerable regional variability. Consequently, it is imperative to devise distinct development methods customized for particular locations.

## 8 Limitations and future directions

This study has made notable contributions. However, there are certain limitations that must be addressed in future research.

First, due to data availability, this study analyzed provincial-level data for China from 2014 to 2023 and used the one-period lag of the sports industry development level as an instrumental variable, applying two-stage least squares (2SLS) to test for endogeneity. Future research can add data from different countries and periods, including international data, in order to examine cross-national comparisons and the temporal lag effects of the sports industry on the health industry.

Second, data accessibility also imposes constraints. Both the sports industry and the health industry are complex systems; limited data access and differences in statistical definitions mean that the evaluation system in this paper primarily follows existing studies and commonly used screening approaches. Because the statistical coverage of the sports industry is incomplete, some variables are proxied by indicators from the “culture, sports and entertainment” category, which may introduce measurement bias and comparability issues. In subsequent research, where data permit, more representative indicators—such as value added and employment in the sports industry—can be included.

Third, this study focuses on R&D intensity as a single mediating variable. Future work can broaden the mechanism map to include digitalization, human capital and professional capacity, health service supply, insurance coverage, and health literacy. The moderating roles of institutional quality, openness, fiscal incentives, and competitive intensity also merit examination. Methodologically, future studies may consider the KHB model, structural equation modeling (SEM), partial least squares structural equation modeling (PLS SEM), and other mediation models with moderation.

Fourth, given that the current development of China's health industry exhibits spatial effects, future research can, on the basis of this study, introduce appropriate methods and models for identifying spatial effects, thereby revealing from a spatial perspective the spillover effects and driving mechanisms through which the sports industry influences the health industry.

## Data Availability

The original contributions presented in the study are included in the article/Supplementary material, further inquiries can be directed to the corresponding author.
